# Enhancing pedestrian perceived safety through walking environment modification considering traffic and walking infrastructure

**DOI:** 10.3389/fpubh.2023.1326468

**Published:** 2024-01-08

**Authors:** Yeonjoo Kim, Byungjoo Choi, Minji Choi, Seunghui Ahn, Sungjoo Hwang

**Affiliations:** ^1^Department of Architectural and Urban Systems Engineering, Ewha Womans University, Seoul, Republic of Korea; ^2^Department of Architecture, Ajou University, Suwon, Republic of Korea; ^3^Division of Architecture, College of Engineering, Inha University, Incheon, Republic of Korea

**Keywords:** pedestrian safety, safety perception, walking environment, traffic, infrastructure, mediation analysis

## Abstract

Urban policies have recently been formulated, following the increasing interest in pedestrian-friendly cities, people-centered safety, and accessibility. Despite the research efforts on physical walking safety, safety evaluations centered on pedestrian perception have been under-reported. Investigating the factors affecting pedestrian subjective safety perception is critical to promoting walking intention because pedestrians forgo walking if they feel unsafe. This study explored the relationship between various walking environmental factors and pedestrians’ psychological perception of safety by surveying 99 pedestrians’ perceptions at nine study sites and conducting a field investigation. Because of the multifaceted nature of pedestrian perception, mediation effect analyses were also conducted to understand the relationship between walking environment factors and perceived safety in depth, considering the role of the perception of traffic characteristics and walking infrastructure. This study found that walking environmental factors closely related to physical safety (e.g., traffic safety facilities and crosswalks) may not greatly contribute to perceived safety and demonstrated that maintaining infrastructure quality is essential for enhancing perceived safety, considering the mediating effect of the perception of infrastructure on perceived safety. The results imply that to improve the walking environment, it is necessary to consider both the physical safety and the perceived safety of pedestrians. This requires comprehensive planning for enhancing traffic safety facilities as well as ensuring user comfort and pleasure through quality infrastructure. This study can provide a basis for enhancing pedestrian-centered safety and promoting residents’ walking intention for public health while increasing their perceptions of safety.

## 1 Introduction

Walking is the most environmentally friendly and equitable means of transportation. Walkability is the foundation of sustainable and equitable cities, and planning for the walking environment should be performed to ensure people enjoy safe, efficient, and pleasant walking (1). The transport and urban policy paradigm has, thus, recently emphasized human-centric safety and accessibility rather than vehicle-centric mobility. Enhancing pedestrian safety is essential, as pedestrians are the most vulnerable road users to potential dangers. Pedestrians’ subjective perception of safety is also critical to encouraging walking because pedestrians forgo walking for pleasure unless they feel safe (2). Therefore, research on environmental improvement measures aimed at increasing pedestrian perceived safety is needed to create a walkable environment devoid of fear and anxiety. Accordingly, factors on pedestrians’ perceived safety should first be investigated.

That walking environment factors, such as obstacles, traffic facilities, and pathway characteristics, affect pedestrian physical safety has been widely reported (3). However, while most research has focused on physical safety, research on the relationship between pedestrians’ perception of safety and walking environmental factors is lacking. By identifying the impacts of walking environmental factors on perceived safety, walking environments can be improved by considering both pedestrians’ physical and perceived safety. Therefore, this study aims to identify the relationship between various walking environmental factors and pedestrians’ perceived safety. In particular, pedestrian safety perception comprises various aspects, such as perceptions of dynamic traffic characteristics (e.g., moving vehicles) and static infrastructure for walking (e.g., traffic facilities and pathway conditions); therefore, the impact of walking environmental factors is analyzed by considering both the role of human perception on traffic and walking infrastructure on perceived safety.

In this study, “perception of traffic,” “perception of walking infrastructure,” and “overall perceived safety” are measured, and linear regression analysis is conducted to investigate the relationship between them. The regression analysis also explores the relationship between walking environmental factors and perceived safety. Finally, the mediation effect of the perception of traffic and walking infrastructure between walking environmental factors and pedestrians’ perceived safety is analyzed to elucidate this relationship.

The scope of perceived safety in this study is confined to pedestrian traffic-related safety, and the research sites are busy streets on and around university campuses located in Seoul’s city center, where pedestrian safety has been a persistent concern. According to the Korea Consumer Agency (4), 23% of pedestrians were at risk from accidents at such locations, which is higher than elsewhere in Korea. The choice of research sites in this study was informed by the high demand for environmental improvements to enhance pedestrian safety.

## 2 Literature review

The physical factors that affect pedestrian safety have been amply researched. Mukherjee and Mitra (5) statistically proved the following to be the leading causes of pedestrian deaths from traffic accidents: the approaching speed of vehicles, vehicular traffic and pedestrian volume at intersections and their interaction, disorderly movement of traffic, presence of a specific land-use type, inefficient planning and design, a wide carriageway, footpath encroachment, and restricted visibility. Yin and Zhang (6) also identified a relationship between pedestrian safety and built environment variables, such as intersection density. Sheykhfard et al. (7, 8) performed pedestrian risk assessment considering the influence of road environment factors, such as transit position, number of lanes, and limited visibility. In addition, many studies on walking environmental factors for vulnerable pedestrians, such as older adults and children, have been conducted. Park and Byeon (3) analyzed the correlation between land usage patterns and pedestrians’ risk from traffic accidents and proposed the management of obstacles, street lighting, traffic signs, and road ratios to lower the risk of traffic accidents around elementary schools. Kim (9) performed regression analysis and found that various facilities, such as raised medians, three-way intersections, street trees, parks, and recreational land use, increased the safety of aged pedestrians. Sheykhfard et al. (10) identified factors affecting the safety of student pedestrians, focusing on crossing behavior at a crosswalk near a university campus. Lv et al. (11) conducted Poisson regression and analyzed the relationship between aged pedestrians and built environments; roads’ green spaces, sidewalks, and intersections significantly affected the safety of these pedestrians, and green spaces only exerted their influence in an uncongested environment. Fonseca et al. (12) also found that numerous built environment attributes affected overall walkability indices, such as residential density and pedestrian facilities. Incheon Metropolitan City (13) studied on-site walking conditions by considering environmental factors, such as sidewalk separation status, total road width, amount of walking, crosswalks, bollards, speed control facilities, traffic signs, speed limit, sidewalk width, sidewalk condition, and obstacles, as walkability improvement indicators.

Although numerous studies on physical environmental factors affect pedestrian safety, research on pedestrians’ perceived safety is somewhat limited despite recent interest in human-centric, pedestrian-friendly, safe, and walkable cities. For instance, Rišová and Madajová (14) measured perceived safety and walkability according to sex and time by dividing spaces and proposed a method of minimizing walking barriers. Park and Garcia (15) explored the relationship between road conditions and pedestrians’ perceived safety in Auburn, Alabama, in the US, and proposed measures to improve public safety perception. Jansson (16) presented social control and urban structure as factors affecting pedestrian safety perception and found that people feel safer in the streets where police or safety personnel are present for social control; the greater the number of people on the streets, the safer they feel. Lee et al. (17) investigated pedestrian perceptions of safety-related information in the walking environment, focusing on individual situation awareness. Ariffin and Zahari (18) concluded that proximity to the destination and good weather conditions promoted walking and increased perceived walkability. Most of these walkability and pedestrian safety studies focused on social and environmental factors. However, few studies have analyzed the impact of various physical environmental factors on pedestrians’ perceived safety. Zumelzu et al. (19) analyzed the impact of the built environment on the perception of walkability but did not specify perceived safety. Villaveces et al.’s (20) study was limited to analyzing the relationship within an entire physical environment, such as an urban structure; the study did not identify each environmental factor. Basu et al. (21) also investigated the influence of built environment factors on pedestrians’ perceptions of attractiveness and safety, but these factors were limited to meso- or macroscale factors, such as land use and landscaping elements, including green areas. Similarly, Amiour et al. (22) systematically reviewed articles on objective and perceived traffic safety, highlighting that only a few papers went into detail on safety perception related to the objective built environment.

The literature review reveals that pedestrian safety perception has been under-researched; because a well-constructed physical environment may not necessarily make pedestrians feel safe, the relationship between walking environmental factors and perceived safety should be further studied to improve both pedestrians’ physical and perceived safety. In this regard, a previous study showed that perceived safety has a significant effect on the choice of walking (23). Because research on safety perception has focused on social and macro- and mesoscale environmental factors that are difficult to manage, micro-scale physical environmental factors that are relatively easy to control need to be further studied. In addition, because people and vehicles coexist on a variety of walking infrastructures, the underlying perceptions of traffic characteristics and infrastructure must be comprehensively considered by analyzing the impact of microscale physical environmental factors and perceived safety. Therefore, the creation and improvement of pedestrian environments and transportation facilities will not only promote the physical safety of pedestrians but also lower pedestrians’ perceptions of danger, making walking the preferred transport mode.

## 3 Materials and methods

### 3.1 Research hypothesis and process

**Figure 1:**
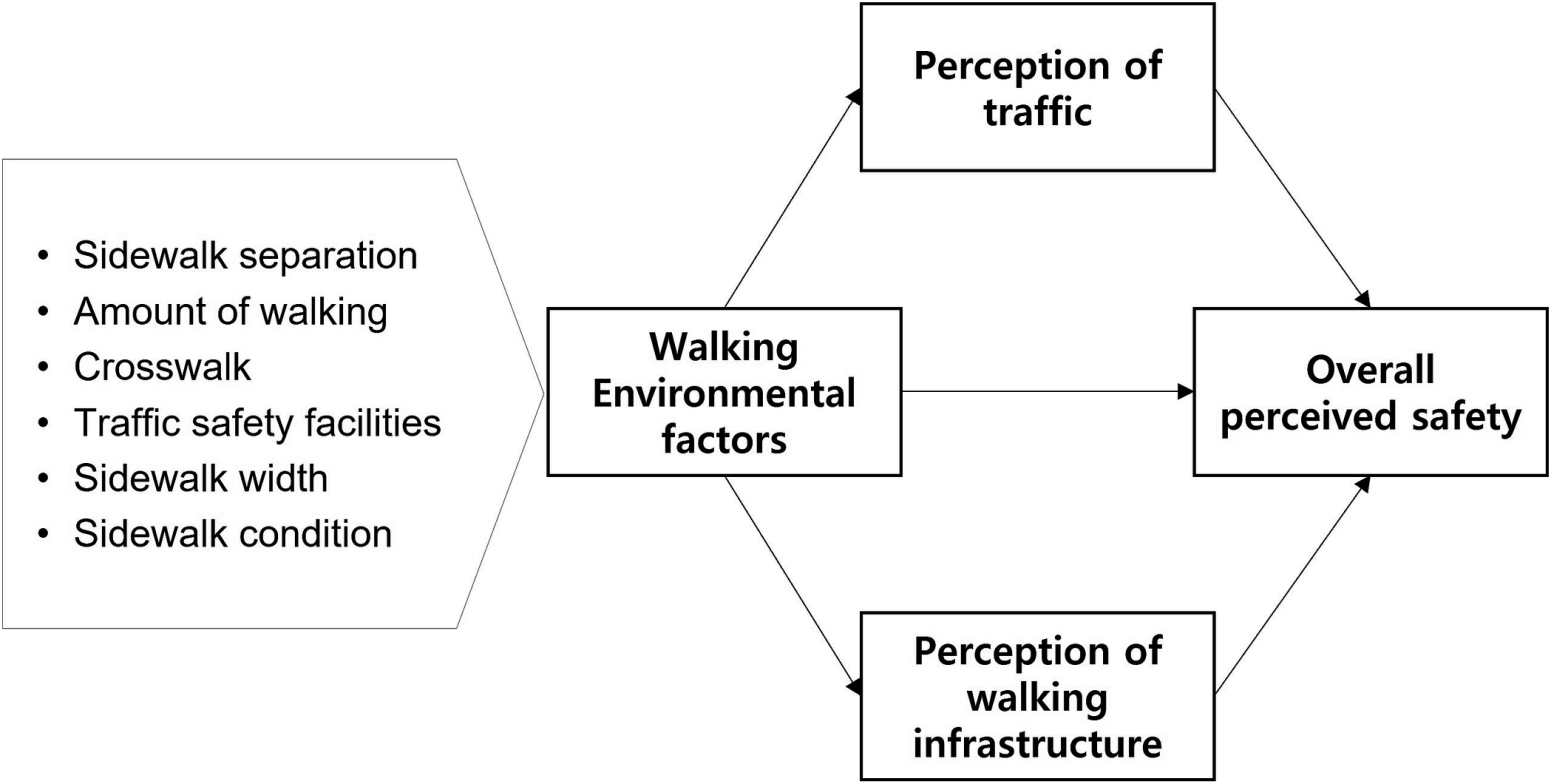
Research model.

Figure 1 shows the research model. This study is based on the hypothesis that the effects of physical walking environment factors on pedestrian safety perception are complex. This complexity may be attributed to pedestrians’ perception having several aspects, such as perception of physical infrastructure as well as traffic or crime hazards (24). As this study focuses on pedestrian traffic-related safety, factors such as crime safety are excluded from the analysis. To elucidate perceived safety, this study was thus based on a model that posits that the two subelements of pedestrians’ perception—perception of traffic and perception of walking infrastructure—analogously mediate pedestrians’ overall perceived safety. Many microscale physical walking environmental factors may impact safety perception. Among many factors, this study chose those that were used as the indicators of walking environment improvement from Incheon Metropolitan City (13) because the indicators provide quantitative evaluation criteria for various walking environment elements and have been used in multiple real-world environments. They include sidewalk separation; amount of walking; number of crosswalks; traffic safety facilities such as speed control facilities and traffic signs and road marks; sidewalk width, and sidewalk conditions considering the obstacles, which are relatively easy to improve or monitor. Some factors are more related to physical safety (e.g., traffic safety facilities), whereas others are more related to mobility or comfort (e.g., sidewalk width and condition). In this study, we hypothesize that each factor will affect sub-dimensions of pedestrian perceptions differently and thus have different effects on overall perceived safety.

To test the hypotheses of the research model in Figure 1, the authors selected study sites with different walking environmental factors and measured the value of walking environmental factors through field investigation. The authors then measured the perception of traffic, perception of walking infrastructure, and overall perceived safety level of each site by surveying participants familiar with the sites. The analysis has three phases. First, linear regression analysis was conducted to determine the impact of perception of traffic and perception of walking infrastructure on overall perceived safety. Multiple regression analysis was then performed to explore the impact of physical walking environmental factors on the research sites. Finally, to elucidate safety perceptions, mediation analysis was conducted to identify whether different pedestrian perceptions—perception of traffic and perception of walking infrastructure—mediate between walking environmental factors and overall perceived safety.

### 3.2 Study sites

**Figure 2:**
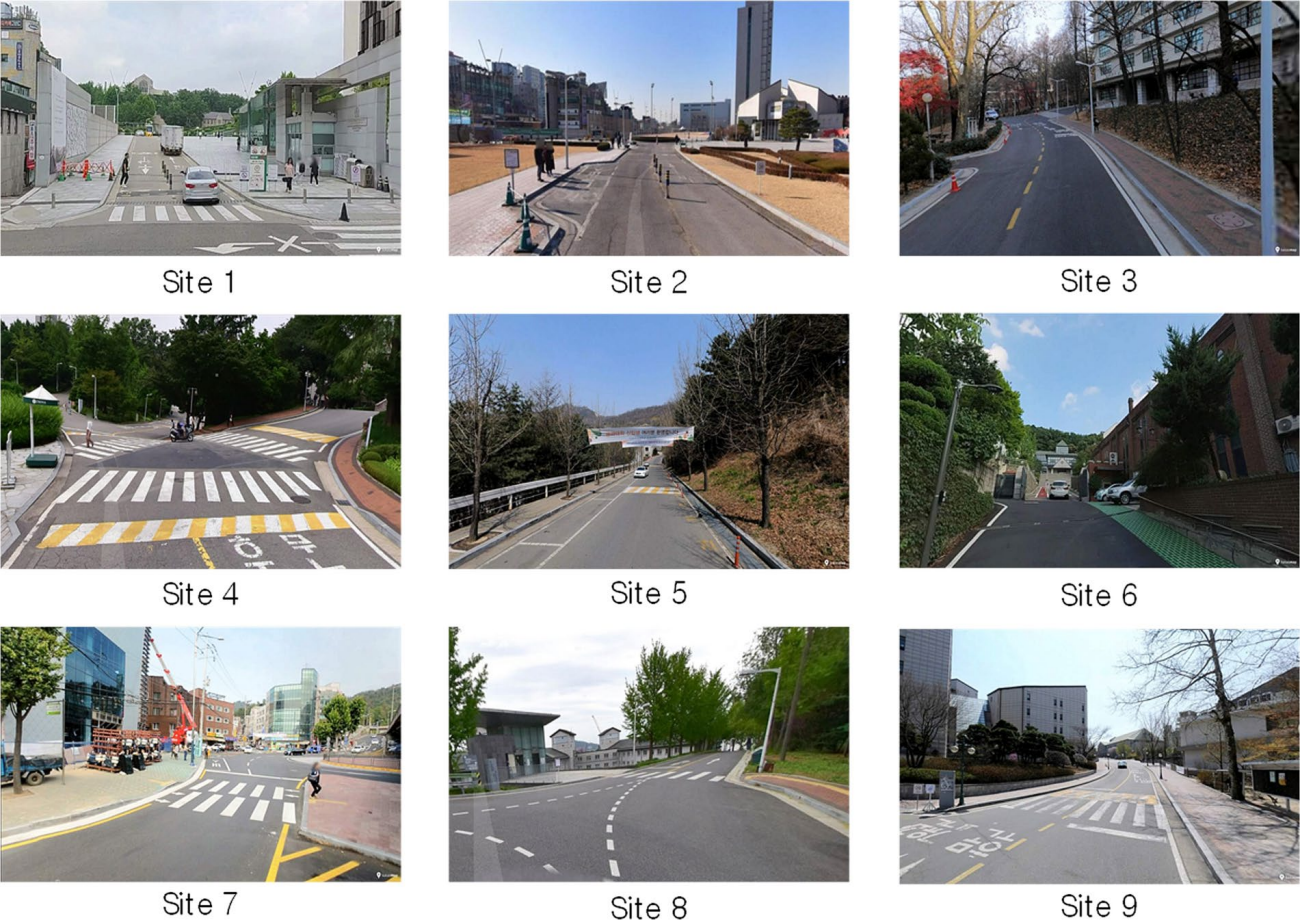
Sample photographs of research sites.

A preliminary site survey was conducted to identify research sites with pedestrian safety problems or high pedestrian traffic. The study sites comprise nine pedestrian roads with various walking environment features. Sites 1 and 2 feature clear sidewalk separations at both sides, good sidewalk conditions, and high vehicular and pedestrian traffic. Site 1 is the entrance of a university with a high traffic volume and the widest sidewalk. Site 3 has clear sidewalk separations at one side and wide roads. Site 4 is an intersection with vehicles moving in multiple directions and has high pedestrian traffic. Site 5 has clear sidewalk separations on one side; however, the width is very narrow, making it difficult for many people to use the sidewalk. Site 6 is a secondary entrance to the aforementioned university with a high traffic volume, unclear sidewalk demarcations, and poor sidewalk conditions. Site 7 has considerable traffic without traffic lights and poor sidewalk conditions. Site 8 is an intersection without clear sidewalk separation. Site 9 has high traffic volumes but is well-equipped with crosswalks and sidewalks. Figure 2 shows sample photographs of each research site.

To further analyze the pedestrians’ perceived safety in each site, physical walking environments and traffic characteristics of each site were examined through an in-depth field study. Table 1 lists the features of each environmental factor for each site. The factors identified by the Incheon Metropolitan City (2022) were used to evaluate the walking environment. They were graded A if the sidewalks on both sides were well separated, B if somewhat separated but the sidewalk was interrupted or installed on only one side, and C if not well separated. The grading was converted to a three-point scale for quantitative analysis in regression analysis. Traffic safety facilities were measured as the combined number of speed control facilities, traffic signs, and road marks. The sidewalk width represented the width of the sidewalk that pedestrians can actually use. The sidewalk condition was evaluated using the sidewalk pavement condition according to Rule 237 of the Guidelines for Sidewalk Installation and Management set forth by the Ministry of Land, Infrastructure, and Transport in Korea. These factors were rated in alphabetical order from A (good) to E (poor) and converted to a 5-point scale for quantitative analysis during regression analysis. The factors were closely related to pedestrian physical safety as well as accessibility, mobility, and comfort.

**Table 1:** Walking environmental factors by sites.

Factors	Unit	Sites								
		1	2	3	4	5	6	7	8	9
Sidewalk separation	A (Good)–C (Bad)	A	A	B	A	C	C	A	C	A
Amount of walking	Person/h	400	400	200	200	30	30	100	50	150
Crosswalk	Number	1	1	4	3	0	0	1	1	3
Traffic safety facilities	Number	7	4	4	4	5	0	0	0	3
Sidewalk width	Meter	9.2	2.83	2.2	3.81	1.02	1.36	5.88	1.78	5.77
Sidewalk condition	A (Good)–E (Bad)	A	A	A	B	B	D	C	B	A

### 3.3 Survey for measuring perceived safety

It is essential to measure the feeling of safety, which reflects the pedestrians’ psychological condition. The Neighborhood Environment Walking Scale (NEWS-A) was consulted to quantitatively measure safety perceptions and identify subscales for deeper analysis (25, 26). NEWS-A is a global survey tool designed to measure how residents perceive their environment. It is widely used in assessing walking environments and walkability and focuses on users’ perceptions, which is closely related to this study. Subquestions for measuring pedestrian perception in the NEWS-A comprised eight categories: residential density, land use mix-diversity, land use mix-access, street connectivity, walking/cycling infrastructure, esthetics, automobile traffic, and crime safety (24), among which perception on walking infrastructure and traffic fall within the scope of this research (i.e., the relationship between microscale physical walking environmental factors and perceived safety). The survey items for this study were also structured accordingly by utilizing the questionnaire provided by NEWS-A. Notably, NEWS-A has been used extensively in walkability studies, making it a highly reliable survey instrument (27).

The survey comprised six questions in total: two on the perception of traffic, two on the perception of walking infrastructure, and two on overall perceived safety (Table 2). The survey was conducted in each study site; it comprised 54 questions in total, 6 for each of the nine sites. Due to the multiplicity of study sites, the three aspects of the safety construct were reduced to two questions to minimize respondents’ fatigue. A five-point Likert scale was used to evaluate the survey responses regarding satisfaction as follows: very satisfied (22), satisfied (27), neutral (2), dissatisfied (4), and very dissatisfied (13). Local community members who were familiar with all the study sites were selected as survey respondents. A link to the online survey was distributed to a local online community with a wide range of ages. The survey was then completed by volunteers who were interested in pedestrian safety in their neighborhood. A total of 99 eligible responses out of 125 were analyzed after excluding 26 unreliable ones. As the research site largely has young and middle-aged people, the respondents were mainly healthy, aged 10–40. An *a priori* power analysis was conducted using G*Power version 3.1.9.7 (28) to determine the minimum sample size required to test the study hypothesis. The results indicated that the sample size required to achieve an 80% power for detecting a medium effect, at a significance criterion of *α* = 0.05, was *N* = 98 for the *F*-test for multiple linear regression with six predictors. Thus, the obtained sample size of *N* = 99 in this study was adequate to test the study hypothesis. To verify the reliability of the results, Cronbach’s alpha was used to indicate the consistency of answers between similar questions. Cronbach’s alpha was at least 0.6 per question, indicating that they were reliable.

**Table 2:** Survey questionnaire.

Constructs	Questions
Perception of traffic	The speed of motor vehicles/motorbikes in this area is appropriate.
	The speed and amount of traffic do not create a sense of danger for pedestrians.
Perception of walking infrastructure	Walkways are well separated from vehicles and motorbikes, and motorcycles.
	Walking facilities such as sidewalks and crosswalks are well arranged.
Overall perceived safety	This area does not feel dangerous for walking.
	I generally feel safe in this area.

## 4 Results

### 4.1 Descriptive analysis of perceived safety by sites

Table 3 presents the survey results for perceived safety in each site. Each site had a minimum score of 1 (bad) and a maximum score of 5 (good) for perceptions of traffic and walking infrastructure, and overall perceived safety. The results revealed that the average score of all sites for the perception of traffic, perception of walking infrastructure, and overall perceived safety were 3.20, 3.07, and 3.03, respectively. The results per site indicated that Site 2, which has clear sidewalk separations and good sidewalk conditions, was perceived as satisfactory in terms of traffic and infrastructure and, thus, safest. In addition, Site 3 had clear sidewalk separations and good sidewalk conditions, which translated to a relatively good safety perception. Site 6, which has frequent traffic, unclear sidewalk separation, and poor sidewalk conditions, was perceived as the least safe, with dissatisfaction in terms of traffic characteristics and infrastructure.

**Table 3:** Survey results for pedestrian perceptions by sites.

	Perception of traffic (1 (bad)-5 (good)) [Mean(SD)]	Perception of walking infrastructure (1 (bad)-5 (good)) [Mean(SD)]	Overall perceived safety (1 (bad)-5 (good)) [Mean(SD)]
Site 1	3.21 (1.18)	3.03 (1.10)	2.79 (1.09)
Site 2	3.88 (0.69)	3.75 (0.83)	3.72 (0.74)
Site 3	3.65 (0.79)	3.45 (0.95)	3.55 (0.89)
Site 4	2.87 (0.97)	2.75 (0.80)	2.66 (0.85)
Site 5	3.27 (0.81)	2.99 (0.89)	3.07 (0.88)
Site 6	2.62 (0.92)	2.44 (0.90)	2.53 (0.84)
Site 7	2.85 (1.06)	2.92 (0.92)	2.70 (0.89)
Site 8	3.00 (0.71)	2.86 (0.75)	2.89 (0.64)
Site 9	3.52 (0.62)	3.44 (0.54)	3.38 (0.62)
Total	3.20 (0.75)	3.07 (0.83)	3.03 (0.89)

A linear regression analysis was conducted to determine whether perceptions of traffic or walking infrastructure affect the overall perceived safety of each site (Table 4). In the analysis by factor of all sites, the significance probability was less than or equal to 0.001, implying that the perceived safety was significant. Both perceptions of traffic and walking infrastructure significantly impacted perceived safety in all sites. A linear regression analysis was performed to determine the impact of perception of traffic and perception of walking infrastructure on safety perception in all nine sites (Table 5). The regression model was statistically significant (*F* = 1110.998, *p* < 0.001), and its power of explanation was approximately 71.4%. The Durbin–Watson (D-W) statistic also had a value of 1735, indicating no independence assumption issues; all VIF values were less than 10, suggesting that multicollinearity issues were absent. The Breusch–Pagan test is used to determine whether or not heteroscedasticity is present in the multiple regression model (29). The results revealed that the null hypothesis for homoscedasticity (i.e., the residuals are distributed with equal variance) cannot be rejected (BP = 2.838, *p* = 0.242), implying that heteroscedasticity was absent in the model. The results demonstrated that both perceptions of traffic and infrastructure had a significant linear relationship with overall perceived safety. Although the perception of infrastructure was slightly more influential, the results imply that pedestrian perception of safety is affected by pedestrian perceptions of dynamic traffic characteristics of pathways, such as the amount and speed of vehicles and physical infrastructure characteristics.

**Table 4:** Regression analysis on the effects of the perception of traffic and walking environment on perceived safety by sites.

	*B*	SE	*β*	*t*	*p*
Site 1					
Traffic	0.751	0.070	0.735	10.690	<0.001
Walking infrastructure	0.792	0.068	0.763	11.625	<0.001
Site 2					
Traffic	0.675	0.100	0.566	6.762	<0.001
Walking infrastructure	0.780	0.069	0.753	11.257	<0.001
Site 3					
Traffic	0.998	0.058	0.869	17.328	<0.001
Walking infrastructure	0.889	0.053	0.864	16.927	<0.001
Site 4					
Traffic	0.833	0.075	0.749	11.122	<0.001
Walking infrastructure	0.955	0.063	0.838	15.125	<0.001
Site 5					
Traffic	0.918	0.077	0.772	11.971	<0.001
Walking infrastructure	0.847	0.061	0.814	13.811	<0.001
Site 6					
Traffic	0.979	0.063	0.845	15.589	<0.001
Walking infrastructure	0.881	0.037	0.924	23.836	<0.001
Site 7					
Traffic	0.720	0.080	0.675	9.003	<0.001
Walking infrastructure	0.925	0.044	0.906	21.097	<0.001
Site 8					
Traffic	0.683	0.073	0.687	9.324	<0.001
Walking infrastructure	0.722	0.063	0.758	11.456	<0.001
Site 9					
Traffic	0.696	0.090	0.616	7.706	<0.001
Walking infrastructure	0.822	0.078	0.730	10.531	<0.001

**Table 5:** Regression analysis on the effects of the perception of traffic and walking environment on the perceived safety of all sites.

	*B*	SE	*β*	*t*	*p*	VIF
Traffic	0.067	0.017	0.072	3.887	<0.001	1.059
Walking infrastructure	0.853	0.019	0.826	44.745	<0.001	1.059

*F* = 1110.998 (*p* < 0.001), *R*^2^ = 0.714, adj R^2^ = 0.714, D-W = 1.735.

### 4.2 Regression analysis between environmental factors and perceived safety

**Figure 3:**
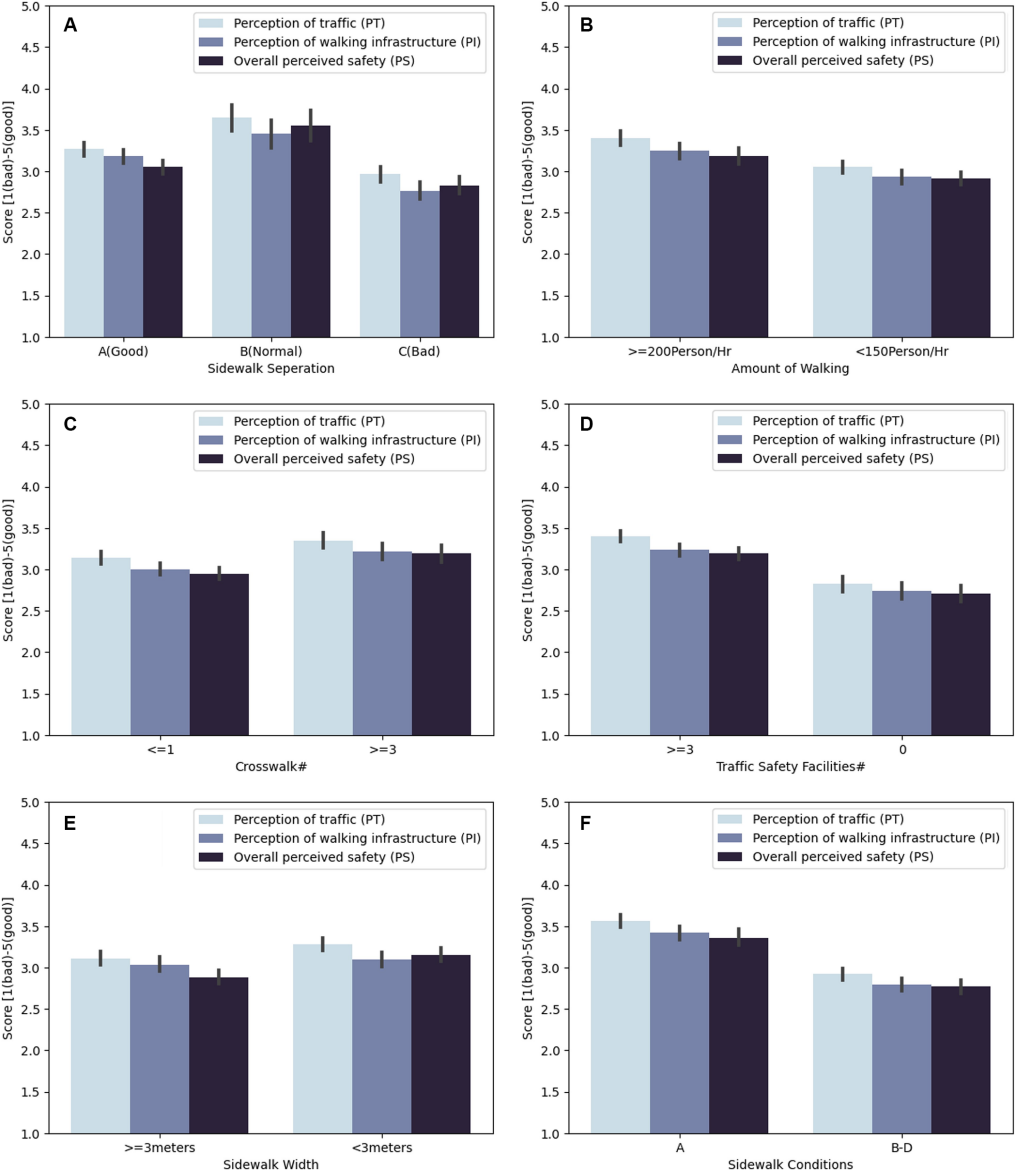
Pedestrian perception according to walking environmental factors: **(A)** The impact of sidewalk separation; **(B)** the impact of the amount of walking; **(C)** the impact of the number of crosswalks; **(D)** the impact of the number of traffic safety facilities; **(E)** the impact of sidewalk with; and **(F)** the impact of sidewalk conditions.

As a preliminary analysis, sites with different walking environmental factors, based on the results from Table 1, were compared with the scores of perception of traffic, perception of walking infrastructure, and overall perceived safety (Figure 3). Figure 3 on safety perception revealed differences between well-equipped and poorly equipped walking environmental factors, such as the degree of sidewalk separation, the number of crosswalks, the number of traffic safety facilities, and the level of sidewalk conditions. The descriptive figure implies that safety perception is generally higher in well-managed locations, with more traffic safety facilities and crosswalks and better sidewalk conditions, in keeping with walking environment improvement plans. On the other hand, when the sidewalk width was greater, the value for the perception of traffic was slightly weaker and likely to be affected by high vehicle and pedestrian flow rates in the busy street. In summary, most environmental factors unsurprisingly exhibited a consistently positive and negative relationship with the perception of traffic, perception of walking infrastructure, and overall perceived safety. However, the sidewalk width exhibited an opposite or insignificant relationship with these measures.

Before further identifying perceived safety-related factors, Pearson correlation was measured to analyze the relationships between the variables (Table 6). It revealed that most variables had positive relationships. In particular, sidewalk separation, speed facilities, and sidewalk condition had a correlation coefficient greater than 0.7, indicating high linear correlations with each other. However, sidewalk width was not significantly related to any pedestrian perceptions. Nevertheless, as shown in Figure 3, too narrow a sidewalk width negatively impacts pedestrian perceptions, and an adequate sidewalk width positively impacts perceptions.

**Table 6:** Pearson correlations between variables.

Correlations(*N* = 594)									
	Sidewalk separation	Amount of walking	Cross-walk	Traffic safety facilities	Sidewalk width	Sidewalk condition	PT	PI	PS
Sidewalk separation	1								
Amount of walking	0.708**	1							
Crosswalk	0.442**	0.227**	1						
Traffic safety facilities	0.360**	0.666**	0.211**	1					
Sidewalk width	0.751**	0.564*	0.148**	0.381	1				
Sidewalk condition	0.461**	0.671**	0.537**	0.712**	0.335**	1			
PT	0.232**	0.312**	0.255**	0.200	0.365	0.365**	1		
PI	0.194**	0.247**	0.177**	0.175**	0.344	0.344**	0.235**	1	
PS	0.090**	0.178**	0.175**	0.146	−0.065	0.317**	0.266**	0.842**	1

**p < 0.01, *p < 0.05. PT, perception of traffic; PI, perception of walking infrastructure; PS, overall perceived safety.

Multiple regression analysis was performed to determine the effects of walking environmental factors on overall perceived safety, and the results are listed in Table 7. The regression model was statistically significant (*F* = 26.023, *p* < 0.001) with 15.0% of the power of explanation. There were no independence assumption issues with 1.491 of D-W statistic and no multicollinearity issues with all VIF values less than 10. The Breusch–Pagan test result (BP = 7.784, *p* = 0.2544) also demonstrated that heteroscedasticity is absent in the multiple regression model.

**Table 7:** Multiple regression analysis of the effect of walking environmental factors on overall pedestrian perceived safety.

	Unstandardized		Standardized	*t*	*p*	VIF
	*B*	SE	*β*			
Sidewalk separation	0.170	0.071	0.165	2.401	0.017*	4.938
Amount of walking	0.000	0.000	0.023	0.361	0.718	4.240
Crosswalk	−0.053	0.032	−0.076	−1.656	0.098	2.205
Traffic safety facilities	−0.048	0.020	−0.121	−2.421	0.016*	2.613
Sidewalk width	−0.109	0.019	−0.296	−5.791	<0.001*	2.709
Sidewalk condition	0.427	0.056	0.451	7.635	0.001*	3.633

*F* = 26.023 (*p* < 0.001), *R*^2^ = 0.150, adj *R*^2^ = 0.144, D-W = 1.491.

A test for the significance of regression coefficients demonstrated that sidewalk separation (*β* = 0.165; *p* = 0.017) and sidewalk condition (*β* = 0.451; *p* = 0.001) had a significant positive impact on perceived safety. Furthermore, traffic safety facilities (*β* = −0.048, *p* < 0.001) and sidewalk width (*β* = −0.296, *p* < 0.001) negatively impacted perceived safety.

Mediation analysis was conducted to verify if the perception of traffic and the perception of walking infrastructure mediate the relationship between walking environmental factors and overall perceived safety (Table 8). To determine direct and indirect effects, the mediation analysis was performed using PROCESS (30), based on the percentile bootstrap method. Specifically, 5,000 samples were obtained via random sampling. Similar to Preacher and Hayes (31), the present study considered the mediation effect to be statistically significant if the lower and upper values of the confidence intervals of the mediation effect coefficients did not contain zero. The mediation analysis demonstrated that the perception of traffic and perception of walking infrastructure mediated the relationship of overall perceived safety with sidewalk separation, amount of walking, crosswalks, speed facilities, and sidewalk condition and did not mediate sidewalk width. In general, the results demonstrate that the mediating effect of the perception of walking infrastructure is significantly larger than that of the perception of traffic or direct effects. Specifically, the indirect impact of the perception of walking infrastructure was relatively higher for sidewalk separation (indirect effect = 0.1669, *p* < 0.05) and sidewalk condition (indirect effect = 0.2678, *p* < 0.05). These findings suggest that creating quality infrastructure from a pedestrian perspective is crucial to perceived safety.

**Table 8:** Mediation analysis results for pedestrian perceived safety.

	Total effect	Direct effects	Indirect effects	Boot SE	Bootstrap CI	
					LLCI	ULCI
Sidewalk separation	0.0928	−0.0956				
Perception of traffic			0.0215*	0.0054	0.0119	0.0329
Perception of walking infrastructure			0.1669*	0.0289	0.1089	0.2229
Amount of walking	0.0012	−0.0004				
Perception of traffic			0.0002*	0	0.0001	0.0003
Perception of walking infrastructure			0.0014*	0.0002	0.001	0.0018
Crosswalk	0.1225	0.0082				
Perception of traffic			0.0123*	0.0037	0.0054	0.0201
Perception of walking infrastructure			0.1020*	0.0192	0.0654	0.1404
Traffic safety facilities	0.0583	−0.0053				
Perception of traffic			0.0059*	0.0018	0.0026	0.0098
Perception of walking infrastructure			0.0577*	0.0112	0.352	0.08
Sidewalk width	−0.0239	−0.0407				
Perception of traffic			0.0004	0.0009	−0.0013	0.0024
Perception of walking infrastructure			0.164	0.0102	−0.0035	0.0364
Sidewalk condition	0.2999	0.0083				
Perception of traffic			0.0238*	0.0071	0.0109	0.383
Perception of walking infrastructure			0.2678*	0.0262	0.2179	0.3198

^*^p < 0.05.

## 5 Discussion

This study found that pedestrians’ overall perceived safety was significantly affected by their perceptions of both traffic characteristics and walking infrastructure, and that perception of walking infrastructure quality particularly seemed to have a deeper relationship with overall perceived safety. Mediation analysis also proved that perception of traffic and perception of walking infrastructure mediated the impacts of all walking environmental factors on overall perceived safety to a different degree, except for the sidewalk width. That is, perceptions of both dynamic traffic characteristics and static infrastructure complexly affect overall perceived safety. Moreover, high indirect impacts of the perception of walking infrastructure, which mediate the relationship of sidewalk separation, crosswalks, traffic safety facilities, and sidewalk conditions with perceived safety, demonstrated that the quality of infrastructure may greatly contribute to enhancing the safety perception. Thus, to create a pedestrian-friendly street where people are actually safe and feel safe, comprehensive management is required, including control over traffic speed and volume and efforts to maintain good infrastructure quality.

Analyzing the effects of the walking environmental factors on perceived safety in detail revealed that the most positive factors were sidewalk separation and sidewalk condition. These factors can enhance the perception of walking infrastructure quality and increase overall perceived safety. As such, the major finding of this study is that factors related to overall pathway quality, such as cleanliness, comfort, and better mobility, can also help increase the pedestrian perception of safety. Keeping sidewalks clean and in good condition is essential to create a walkable city with a feeling of safety. Considering that sidewalk separation, related to both comfort and safety, also turned out to be a significant factor, it is important to separate sidewalks from roads through elevation or fixed bollards to improve perceived safety and encourage walking.

This research finding is in line with the findings of previous studies. Basu et al. (21) claimed that perceptions of attractiveness (or satisfaction) and safety in the built environment need to be considered together even though the study focused on crime security. One of the study’s findings is that pedestrians feel that the walking environment is not only more attractive but also safer when trees are present on the walking path, which highlights the importance of infrastructure quality in enhancing perceived safety. Herrmann-Lunecke et al. (32) also demonstrated that micro-scale elements in the built environment, such as the presence of sidewalks and their cleanliness and quality, could improve pedestrian comfort and safety, which agrees with the results of the current study.

However, the walking environment factors for physical safety do not necessarily have a positive relationship with feelings of safety. Multiple regression analyses revealed that crosswalks and traffic safety facilities had weak negative relationships with perceived safety, which is also supported by the fact that perception of traffic mediated these walking environmental factors and overall perceived safety. These facilities are essential to physical safety. However, these facilities are located in areas with high vehicle traffic, and numerous vehicles lead to a negative perception of traffic and, consequently, less safety perception, as demonstrated in the research result. While it is necessary to place crosswalks and traffic safety facilities on these roads to enhance safety, their management is essential to keep the quality of the roads high and maintain appropriate levels of pedestrians’ feeling of safety. Sidewalk width also negatively impacted the overall perceived safety, which may be because sidewalks are wider in areas with high traffic main roads. While sidewalks being too wide does not have a positive impact on perceived safety, too narrow a sidewalk width has a significant negative impact on pedestrian perception of infrastructure and perceived safety, as shown in Figure 3. Therefore, ensuring adequate sidewalk width is essential. In sum, walking environmental factors closely related to physical safety may not contribute as much to perceived safety in some cases (e.g., traffic safety facilities and crosswalks), and in other cases, factors related to comfort or satisfaction may contribute greatly to perceived safety (e.g., sidewalk conditions). The result suggests that perceived safety is a composite function of physical safety and comfort due to infrastructure quality.

Sites 2 and 3 had the highest perceived safety, whereas Sites 4 and 6 had the lowest. Sites 2 and 3 both had well-managed sidewalk conditions, consistent with the aforementioned results. Site 4 was located at the intersection of four roads with interrupted sidewalks; despite its high traffic and safety blind spots, as the crosswalks were placed in a poorly separated area, they were underused by pedestrians. Moreover, the sidewalk condition in Site 4 was relatively poor (B grade), which led to a low overall perceived safety despite the bollards and crosswalks. This site will require regular sidewalk maintenance and clear sidewalk separation to improve people’s awareness and comfort and separate vehicles and pedestrians. Site 6, with a very low level of perceived safety, had poor sidewalk conditions (D grade) and no clear sidewalk separation. Therefore, the use of sidewalks was limited as they did not function well, as determined by the authors’ field investigation. Site 6 exhibited poor sidewalk conditions, which highlights the need for regular pavement on the sidewalks. Furthermore, ensuring sidewalk separation by raising the sidewalks at Site 6 will help secure physical and perceived safety as well as pedestrian comfort.

In summary, the study confirmed that both perceptions of traffic characteristics and infrastructure affected pedestrians’ perceived safety. Because environmental factors for advanced physical walking safety do not always guarantee pedestrian perceived safety, traffic safety features, such as crosswalks, traffic safety facilities, and sidewalk width, should be considered together with infrastructure quality factors, such as sidewalk maintenance and clear physical and psychological separation from vehicles. The novelty of this study lies in its consideration of the impact of various environmental factors on perceived safety in traffic-related contexts, revealing that these factors affect physical safety and feelings of safety differently. In contrast, many previous studies only address perceived safety in terms of crime safety or security. The main contribution of this study to traffic-related safety is the finding that perceived safety is determined by comprehensively considering physical safety, comfort, and satisfaction on the road. Accordingly, practical ways to improve the pedestrians’ walking environment to promote both physical safety and the perceived safety of pedestrians are suggested. In other words, because comfort and satisfaction greatly affect the feeling of safety, a comprehensive improvement plan is necessary to enhance perceived safety by maintaining the quality of infrastructure through continuous pathway maintenance and removing unpleasant elements while installing traffic safety facilities for physical safety. In this regard, in Crime Prevention through Environmental Design practice, one of the important principles for better-perceived safety is promoting a positive and pleasant environmental image and routine maintenance of the built environment (33), consistent with the research findings.

## 6 Conclusion

As urban safety and livability in walkable communities become increasingly important, city and transportation policies are being formulated to emphasize human-centric safety and accessibility. This trend requires more in-depth research into pedestrians’ safety perception because residents’ feeling of safety is a critical factor for enhancing their walking intention. This study analyzed the relationship between walking environments and pedestrians’ perceived safety. This pedestrian-oriented study considered various aspects of pedestrians’ perceptions of traffic and infrastructure to elucidate their perceived safety; it demonstrated that walking environmental factors for improving physical safety may not have the same effect on improving perceived safety and demonstrated that maintaining infrastructure quality is essential for enhancing perceived safety when considering the role perception of infrastructure has in mediating the relationship between environmental factors and perceived safety. The results suggest that both the physical safety and perceived safety of pedestrians should be considered to improve the walking environment through comprehensive planning for enhancing physical safety through traffic safety facilities. Moreover, user comfort and satisfaction should be ensured through infrastructure quality assurance. Doing so can improve the city’s walkability and residents’ walking intention for public health while increasing perceptions of safety, pleasure, and comfort.

This study has significance in that it reinforces the knowledge base of the impacts of walking environmental factors on perceived safety to help enhance both physical and perceived safety. However, the research sites were limited to nine areas. Furthermore, it included only some of the walking environmental factors due to a multicollinearity issue resulting from a strong correlation of many variables of walking environmental factors (e.g., bollards and traffic signs). The study was limited to locations that are not often used by older adults. Therefore, future studies may expand research areas and walking environmental factors to validate the usability and transferability of the developed model.

## Data availability statement

The raw data supporting the conclusions of this article will be made available by the authors, without undue reservation.

## Ethics statement

The requirement of ethical approval was waived by Ewha Womans University Institutional Review Board for the studies involving humans because the study was conducted based on an anonymous online survey of unspecified participants. The studies were conducted in accordance with the local legislation and institutional requirements. The ethics committee/institutional review board also waived the requirement of written informed consent for participation from the participants or the participants’ legal guardians/next of kin because the study was conducted based on an anonymous online survey of unspecified participants.

## Author contributions

YK: Conceptualization, Data curation, Formal analysis, Methodology, Visualization, Writing – original draft. BC: Formal analysis, Methodology, Software, Validation, Writing – review & editing. MC: Conceptualization, Formal analysis, Writing – review & editing. SA: Investigation, Writing – original draft. SH: Conceptualization, Formal analysis, Funding acquisition, Methodology, Project administration, Resources, Supervision, Writing – review & editing.
